# Evidence for diversifying selection of genetic regions of encoding putative collagen-like host-adhesive fibers in *Pasteuria penetrans*

**DOI:** 10.1093/femsec/fiy217

**Published:** 2018-10-30

**Authors:** Arohi Srivastava, Sharad Mohan, Tim H Mauchline, Keith G Davies

**Affiliations:** 1 Department of Biological and Environmental Sciences, University of Hertfordshire, Hatfield, AL10 9AB, UK; 2 Division of Nematology, Indian Agricultural Research Institute, Pusa Campus, New Delhi 110012, India; 3 Department of AgroEcology, Rothamsted Research, Harpenden, AL5 2JQ, UK; 4 Division of Biotechnology and Plant Health, Norwegian Institute of Bioeconomy Research, Postboks 115, Ås-1431, Norway

**Keywords:** BclA, hair-like nap, endospore, adhesion, bacterial collagens, pathogenicity

## Abstract

*Pasteuria* spp. belong to a group of genetically diverse endospore-forming bacteria (phylum: Firmicutes) that are known to parasitize plant-parasitic nematodes and water fleas (*Daphnia* spp.). Collagen-like fibres form the nap on the surface of endospores and the genes encoding these sequences have been hypothesised to be involved in the adhesion of the endospores of *Pasteuria* spp. to their hosts. We report a group of 17 unique collagen-like genes putatively encoded by *Pasteuria penetrans* (strain: Res148) that formed five different phylogenetic clusters and suggest that collagen-like proteins are an important source of genetic diversity in animal pathogenic Firmicutes including *Pasteuria*. Additionally, and unexpectedly, we identified a putative collagen-like sequence which had a very different sequence structure to the other collagen-like proteins but was similar to the protein sequences in Megaviruses that are involved in host-parasite interactions. We, therefore, suggest that these diverse endospore surface proteins in *Pasteuria* are involved in biological functions, such as cellular adhesion; however, they are not of monophyletic origin and were possibly obtained *de novo* by mutation or possibly through selection acting upon several historic horizontal gene transfer events.

## Teaser

Collagen-like genes that are expressed on the surface of spores are hypothesised to have been obtained by horizontal gene transfer and are involved in the spores' adhesion to nematode cuticle.

## INTRODUCTION

*Pasteuria penetrans* is a parasite of root-knot nematodes (*Meloidogyne* spp). It is one of a number of species of Gram-positive bacteria that form endospores and has the potential to be used as an environmentally benign biological control agent of plant-parasitic nematodes (Davies 2009; Davies *et al*.^2018^; Stirling 2014). A major constraint in its use as a bio-pesticide is its restricted host range; one population of *P. penetrans* will attach to and infect one population of root-knot nematode but not another (Stirling 1985; Davies, Kerry and Fylnn 1988; Espanol *et al*. 1997). The taxonomy of the *Pasteuria* group of bacteria is confused; this is because of its obligate nature and early characterisation had to rely on an isolate's biology; essentially, its life-cycle, morphology and in particular, its host range. However, molecular techniques, including genomics and proteomics, are today playing an increasingly important and crucial role in characterising its diversity.

Currently, three species of *Pasteuria* have been characterised that parasitize plant-parasitic nematodes: namely *P. penetrans* (Sayre and Starr 1985) on hosts of *Meloidogyne* spp.; *Pasteuria nishizawae* (Sayre *et al*. 1991) on hosts of cyst nematodes *Heterodera* and G*lobodera* spp.; *Pasteuria thornei* (Starr and Sayre 1988) on hosts of *Pratylenchus*, and one *Pasteuria ramosa* (Metchnikoff 1888) that is a parasite of Cladocerans such as *Daphnia*, the water flea. Two other hyperparasites of plant-parasitic nematode species have been characterised and given *Candidatus* status: *Pasteuria usgae* isolated from *Belonolaimus longicaudatus* (Giblin-Davis *et al*. 2003) and *P. aldrichii* (Giblin-Davis *et al*. 2011) on *Bursilla* spp. (Stackebrandt 2014). Two other populations of *Pasteuria* have been characterised, *P. hartismerei*, a parasite of *Meloidogyne ardenensis* and *Pasteuriagoettingianae*, a parasite of *Heterodera goettingiana* but these names, according to Stackebrandt (2014) are invalid.

Early molecular methods of *Pasteuria* characterisation tended to focus on using 16S rRNA genes to differentiate species and populations (Sturhan 1988; Anderson *et al*. 1999; Duan *et al*. 2003). Interestingly, a closely related population of *Pasteuria* isolated from an Indian plant-parasitic cyst nematode *Heterodera cajani* population (Sharma and Davies 1996) has recently been shown to be more promiscuous; endospores of this particular *Pasteuria* population attach to and infect not only the original cyst nematode host, *H. cajani*, but also nematodes from the genus *Globodera* (Mohan *et al*. 2012); more recent results using 16S rRNA showed that it was closely related to *P. nishizawae* with 98.6% base-pair similarity whilst endospores of *P. nishizawae* from *Heterodera glycines* (soya bean cyst nematode) also adhered to a range of cyst nematodes, including *Heterodera espedezaei* (Lespedezae cyst nematode), *Heterodera schachtii* (sugar beet cyst nematode), *H. trifolii* (clover cyst nematode) and *Globodera rostochiensis* (potato cyst nematode), but unlike the *H. cajani* population of *Pasteuria* from India, they neither infected nor complete their life cycle in any nematode other than *H. glycines* (Sayre *et al*. 1991; Atibalentja, Jakstys and Noel 2004; Noel, Atibalentja and Domier 2005). It would, therefore, appear that the relatedness of these *Pasteuria* from *H. cajani* and *H glycines*, as constructed with 16S rRNA, does not reflect host range. This is consistent with endospore attachment studies of root-knot nematodes where adhesion was not linked to nematode phylogeny (Davies *et al*. 2001).

Ribosomal RNA (16S rRNA) was used for the reconstruction of the ‘tree of life’ (Woese 1987) and it has been universally accepted as a tool for phylogenetic reconstruction and classification of prokaryotes. Apart from the known conservation of 16S rRNA at the level of nucleotide sequences and secondary structures, the classical concept presumed that these genes do not undergo horizontal gene transfer (HGT) events. However, several studies report the horizontal transfer of segments of this gene and advocate the need for alternative methods to avoid misidentification and interpretation of discordant phylogenies (Eardly, Wang and Van Berkum 1996; Yap, Zhang and Wang 1999; Schouls, Schot and Jacobs 2003; Rajendhran and Gunasekaran 2011). Protein-coding housekeeping genes may have advantages over ribosomal RNA genes and a multilocus protein sequence approach, using 25 or more housekeeping genes translated from a genome survey sequence of strain RES147, produced a robust bacterial phylogeny that suggested the *Pasteuria* genus was ancestral to *Bacillus* (Charles *et al*. 2005); additionally, single nucleotide polymorphisms in protein-encoding genes provided increased phylogenetic discrimination than using 16S rRNA sequences (Mauchline *et al*. 2011).

Although these protein-coding genes may be more useful in determining the phylogenetic relatedness of *Pasteuria* between closely related Firmicutes than 16S RNA, they are unlikely to relate in any meaningful manner to host-range and pathogenicity.

Another protein-encoding gene with potentially the ability to characterise endospore-forming Firmicutes that have been found in animal parasitic bacteria is *bclA*. This gene, originally characterised in *Bacillus anthracis*, is expressed during the final stages of endospore formation and produces a collagen-like protein (CLP) on the surface of the endospore's exosporium which forms a hair-like nap (Sylvestre, Mock and Couture-Tosi 2002; Steichen *et al*. 2003; Sylvestre, Couture-Tosi and Mock 2003; Todd *et al*. 2003). Genome survey sequences of *P. penetrans* have revealed that they too contain collagen-like genes (Davies and Opperman 2006) and it has been hypothesised that the expression of these collagen-like genes produces a similar hair-like nap on the surface of the *Pasteuria* endospore that is involved in a *Velcro*-like attachment process (Davies 2009). More recently, a large family of polymorphic collagen-like genes have been described in *P. ramosa*, the endospore-forming parasite of *Daphnia* spp. (Mouton *et al*. 2009; McElroy *et al*. 2011) and they are possibly involved in the interaction between *P. ramosa* and its *Daphnia* host (Luijckx *et al*. 2011; Luijckx *et al*. 2013).

As attachment of *P. penetrans* endospores to nematodes has been shown to be unrelated to nematode phylogeny (Davies *et al*. 2001) we would hypothesise that the phylogenetic relatedness using protein-encoding house-keeping genes of *P. penetrans* would be very different from the phylogenetic relatedness described by a protein involved in a key aspect of pathogenicity like endospore adhesion to its potential host. The present investigation uses *in-silico* comparative bioinformatics to look at the phylogenetic relatedness between *P. penetrans* and other selected closely related Firmicutes based on their 16S rRNA phylogeny and to compare this to the phylogenetic trees using the low-complexity collagen-like repeat regions.

## MATERIALS AND METHODS

### Search for protein sequences coding for putative collagens in *Pasteuria*

Collagen-like genes were predicted in unpublished contigs for *Pasteuria* Res148 isolate, a related but host-selected sub-population, of Res147 (Mauchline *et*. 2011).

Gene predictions were done using the RAST annotation web server (Aziz *et al*. 2008) and the annotations were searched for any predicted collagen-like sequences based on the comparison of contig annotations with the sequenced genome of a closely related Firmicute, *Bacillus thuringiensis* Al Hakam. To look for any collagen-like sequences not predicted by RAST, the contigs were uploaded on to Artemis genome browser and annotation tool (Carver *et al*. 2012) and were manually searched for open reading frames containing ‘G-X-Y’ triplet amino acid repeats using the ‘navigator’ feature of Artemis (Rutherford *et al*. 2000) (Rutherford *et al*. 2000). The sequences were further tested for the presence of collagen-like motifs using MOTIF search tool of GenomeNet web server Kanehisa *et al*. (2002).

### Comparison of CL sequences in *Pasteuria* and other collagens

To search for similar sequences in other organisms, the putative *Pasteuria* collagens were used as query sequences for BLASTp searches targeting non-redundant (nr) protein database. The low complexity filter was turned on for these searches to avoid any random hits to low complex G-X-Y repeats of collagens. BLASTp hits with significantly low E-values ranging from 0 to 2e^−128^ were pooled together. The G-X-Y repeat regions of selected putative CLPs (File I,Supporting Information) were analysed for the diversity in their percentage amino acid composition. The G-X-Y repeat regions were extracted from the sequences and a customised script was written in *R* (R Development Core Team 2010) to do the following analyses. The Manhattan distance (Kaufman and Rousseeuw 1990) between the percentage amino acid compositions for all possible pairs of sequences was computed. The resulting distance matrix was subjected to an agglomerative hierarchical clustering method using hclust function (Murtagh and Legendre 2011). The heatmap.2 function of gplots package in R (http://cran.r-project.org/web/packages/gplots/index.html) was used to generate a heatmap representation of the Manhattan distances between the percentage amino acid composition of the G-X-Y repeat regions of different collagen-like sequences. The custom R-script used for generating the heatmap can be found in the supplementary material (File II,Supporting Information for the script).

### Conventional molecular phylogenetic tree based on 16S rRNA

The publicly available 16S rRNA sequences of the cladoceran parasitic *P. ramosa* and nematode parasitic *Pasteuria* spp. were compared with selected 16S rRNA sequences of *Clostridium* spp., *Paenibacillus* spp. and *Pelosinus* spp. and three major animal pathogenic *Bacillus* spp. (*B. anthracis*, *B. thuringiensis* and *B. cereus*) and the non-pathogenic *Bacillus subtilis*. Cyanobacterial species including *Microcystis elabens*, *Arthrospira platensis*, *Cyanobium gracile* and/or *Gloeothece* spp. were used as outgroups. The list of accession numbers of sequences used in the analyses can be found in the File III, Table I (Supporting Information) linked to this article (see).

As a standard comparator a phylogenetic tree was constructed using the classic 16S rRNA gene sequences in MEGA7 (Kumar, Stecher and Tamura 2016) using the Maximum Likelihood method based on Tamura-Nei model and JTT matrix-based model for nucleic acid and protein sequences respectively (Jones, Taylor and Thornton 1992; Tamura and Nei 1993). The initial tree was made using BioNJ algorithm (Gascuel 1997). The bootstrap consensus tree was inferred from 500 replicates (Felsenstein 1985).

## RESULTS

### Molecular phylogeny based on the 16S rRNA gene

**Figure 1.**
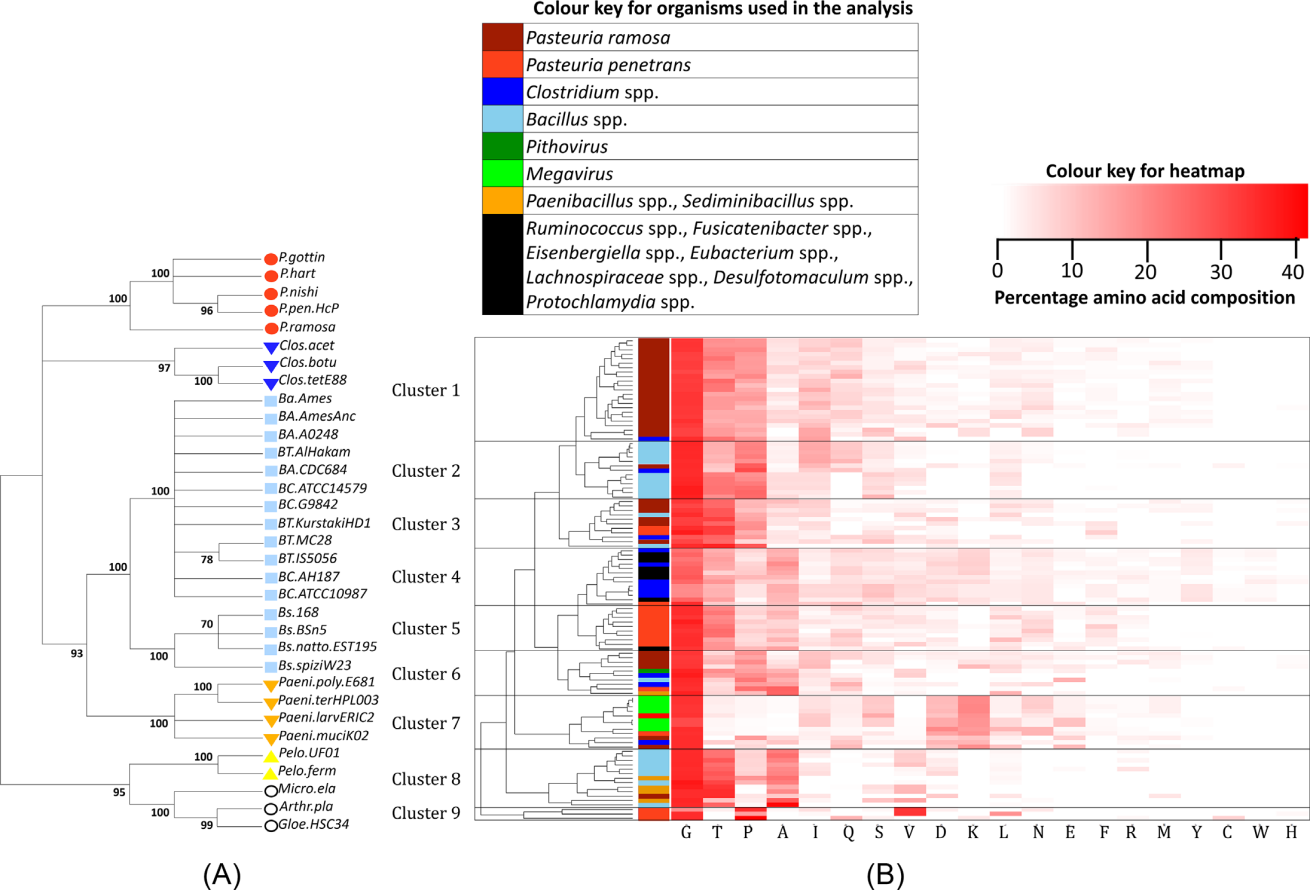
Molecular phylogeny of *Pasteuria* (**A**) The bootstrap consensus tree based on 16S rRNA gene sequences reconstructed using the Maximum Likelihood method. Numbers shown next to the branches are the percentage of replicate trees in which the associated taxa clustered together in the bootstrap test (500 replicates). Only bootstrap values 70% are shown. (**B**) Heatmap reconstructed using the cluster analysis of G-X-Y repeat regions of Ppcl sequences of *P. penetrans*, Pcl sequences of *P. ramosa* and other selected CLPs based on the percentage amino acid composition of the low complex G-X-Y repeat regions.

The phylogenetic tree with the highest log likelihood (-11 556.6217) is shown in Fig. 1a. The analysis involved 33 nucleotide sequences. There was a total of 1821 positions in the final dataset. The 16S rRNA gene sequences from all the five-analysed species of the genus *Pasteuria*, including the cladoceran parasite *P. ramosa*, clustered together with high bootstrap support (100%). *Pasteuria* spp. were observed to be more closely related to *Clostridium* spp. than *Bacillus* species. However, their close-relatedness with *Clostridium* spp. was supported with a low bootstrap value of only 58%. All the *Bacillus* spp. and *Paenibacillus* spp. were grouped together supported by a high bootstrap value. The pathogenic *Bacillus* spp. i.e. *B. cereus*, *B. thuringiensis* and *B. anthracis* were separated from the non-pathogen *B. subtilis* with 100% support. In 95% of the tree replicates, *Pelosinus* spp. was separately grouped as an outgroup with the cyanobacteria.

### Putative collagen-like proteins in *Pasteuria*

Using the sequence-based comparison tool of RAST, 17 putative collagen coding genes were identified in different contigs obtained from the sequencing of *Pasteuria* Res148. Using a manual search, further 16 open reading frames containing G-X-Y repeat regions were found. These sequences (33 in total) were named as Ppcl for ‘*Pasteuria penetrans*collagen-like sequences’ and were numbered as Ppcl1 to Ppcl33. Out of these, 23 sequences were unique and intact (i.e. started with a start codon and ended with a stop codon). To confirm that these sequences are related to the collagen superfamily, these sequences were searched for collagen motifs in MOTIF search. Only 17 of these sequences hit the Pfam: collagen family and were considered as the putative CLPs in *P. penetrans* Res148 (Tables 1 and 2). These 17 Ppcl sequences were selected for further analyses. The G-X-Y repeat regions in some of these putative sequences were interrupted with one or more amino acids, while G-X-Y repeats in eight of the sequences were uninterrupted. See File I (Supporting Information) for the nucleic acid sequences of the 17-selected putative CLPs in *P. penetrans* Res148.

**Table 1.** List of 17 putative CLPs in P. penetrans Res148 (File I, Supporting Information for the complete sequences).

Putative collagen	Length	N-terminal	C-terminal	Number of G-X-Y repeats	Interruptions within the G-X-Y repeat regions and their location within the region
Ppcl1	1215 bp/ 405 aa	MSNLELLHRLCC	RQVVVIELPSGN	83	CVCPP (7..11) CVCPP (39..43)
Ppcl8	753 bp/ 251 aa	MPNHSGLRGSPL	GFVGLVENRGGL	30	SPV (4..6)
Ppcl9	669 bp/ 223 aa	MISVVVTMTSPL	SRSPHAEMDYLP	14	TPVTPVIPVIPVIPVIPV (7..24) DPVAP (28..32) V (36) NPVNPV (46..51) DPV (64..66) NPV (73..75)
Ppcl16	606 bp/ 202 aa	MYHNDYQGKMSD	PCPPPPYPHREY	28	*None*
Ppcl17	1242 bp/ 414 aa	MKRSTKYPFLAM	GQAANLIIRRVF	20	ST (25..26)
Ppcl18	1170 bp/390 aa	MKIKTLLLFILG	TTSISMYVRQIA	23	*None*
Ppcl19	1143 bp/ 381 aa	MIMKAILNIYLI	TAASLLIKRIAS	27	NLQT (73..76)
Ppcl20	780 bp/ 260 aa	MRGNARIGGNLI	RATASVMIRQIF	28	*None*
Ppcl21	1938 bp/ 646 aa	MLEFHLPESSYI	SSGASFTIRRVA	129	IT (19..20)
Ppcl23	1158 bp/ 386 aa	MLAVLLSAPLCA	SISASVLVRRIA	26	*None*
Ppcl24	1203 bp/ 401 aa	MNEVTQLSQADY	GTAFSLMIRRLN	34	*None*
Ppcl25	1179 bp/ 393 aa	MKKIIIYLLLIS	SINASILIRQIS	15	*None*
Ppcl26	837 bp/ 279 aa	MASLNKVRVQLL	TATQANLFFKLV	15	*None*
Ppcl28	843 bp/ 281 aa	MILNLFPPCGFP	VTITKYSDSICS	26	*None*
Ppcl29	837 bp/ 279 aa	MILNLFPPCGFP	VFQYSTNICISQ	29	TFT (79..81)
Ppcl30	774 bp/ 258 aa	MLIGGNLFVNGT	GTAFSLTIIRLN	30	I (3) IT (14..15)
Ppcl33	1770 bp/ 590 aa	MSRSQNNIINYV	SQKTWILIEQIY	49	AEK (124..126)

**Table 2.**
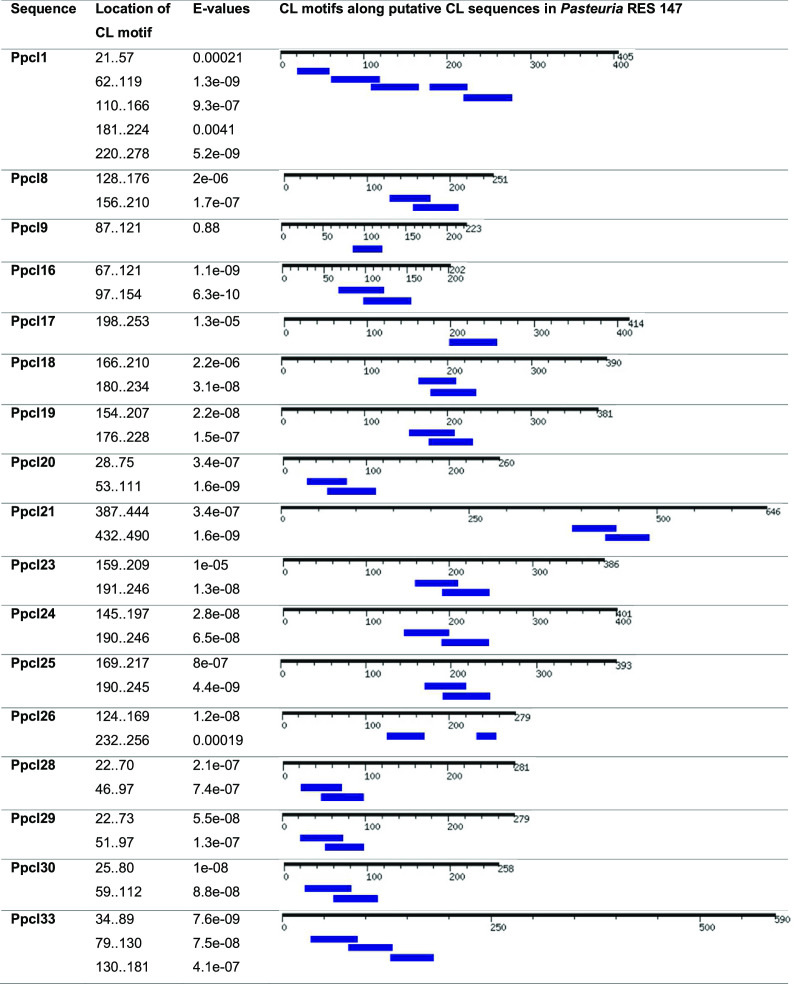
CL motifs in putative collagen-like sequences in *P. penetrans* Res148. CL motifs were predicted by GenomeNet MOTIF using the Pfam database. The location of each CL motif within the sequence is shown along with the E-value for each motif.

### CL sequences similar to *Pasteuria* Ppcl sequences

In the BLASTp searches using putative CL sequences from *P. penetrans*, significant matches were found to Ppcl23, Ppcl25, Ppcl26 and Ppcl33. Ppcl23 hit CL sequences from *Bacillus* spp. (98 hits) *Clostridium arbusti* (1 hit) and *Sediminibacillus albus* (1 hit); all hits were in the E-value range of 0.008 to 3e^−08^. Ppcl25 had 108 BLASTp hits (E-value ≤1e^−07^) from *Bacillus* spp. (96 hits), *Paenibacillus* spp. (7 hits), *Clostridium* spp. (4 hits), *Pithovirus sibericum* (1 hit). All the hits to Ppcl23 and Ppcl25 were in the CTDs of the sequences (query cover: ≤43% for Ppcl23; ≤49% for Ppcl25). Ppcl26 hit CL proteins from a range of bacterial genera including *Clostridium* spp., *Ruminococcus torques*, *Fusicatenibacter* spp., *Eisenbergiella* spp., *Desulfotomaculum* spp., *Lachnospiraceae* spp., *Eubacterium dolichum*, *Blautia producta*, *Bifidobacterium* spp., *Lactonifactor longoviformis*, *Epulopiscium* spp., *Bacteroides gallinarum*, *Parabacteroides* spp., *Methanobrevibacter* spp., *Lactobacillus* spp., *Flavonifractor plautii*, *Leuconostoc* spp., *Veillonella dispar*, *Prevotella* spp. tc2–28. The topmost hits were for proteins from *Clostridium* spp. that had ≥90% query cover and very low E-values ≤1e^−62^. Ppcl33 showed sequence similarity with *Megaviruses* and *Ruminococcus* spp. (E-value: 0 to 2e^−128^; as high as 99% identity for 77% query cover).

### Characterisation of the low-complex G-X-Y repeat region

Of all the significant BLASTp hits to the Ppcl sequences, 52 CL sequences, which showed better query coverage and also had significantly low E-values (in the range of 0 to 2e^−128^), were selected for further analysis, along with 37 previously characterised CL sequences from *P. ramosa*, the BclA and ExsJ protein sequences from *B. anthracis* and *B. cereus*. See File III, Table II (Supporting Information) for the list of accession numbers of the CLP sequences selected for the comparative studies with *Pasteuria* Ppcl sequences. A total of 108 sequences including 17 Ppcl sequences were analysed for the percentage amino acid composition of the G-X-Y repeat regions. It was evident that after Glycine (G), Threonine (T) is the most commonly occurring amino acid, followed by Proline (P). However, other amino acids like Alanine (A) and Valine (V) also occur. The heatmap generated by the hierarchical clustering of the G-X-Y repeat regions is shown in Fig. 1b. The tree splits the sequences categorically into nine clusters.

The Ppcl sequences from *P. penetrans* were spread across five clusters (Cluster 3, 4, 5, 6, 7, 9). Ppcl28 and Ppcl29 clustered together (Cluster 3) with other collagen-like sequences from *P. ramosa* (Pcl28, Pcl17, Pcl8, Pcl18, Pcl32, Pcl30), *Clostridium* spp. (Clos12), *B. thuringiensis* (Bt4) and the BclA protein of *B. anthracis*. Ppcl26 was placed in Cluster 4 along with six collagen-like sequences from *Clostridium* (Clos10, Clos8, Clos7, Clos6, Clos4) and one sequence each from *Fusicateniacter* (Fusica), *Ruminococcus* (Rumino1), *Eisenbergiella* (Eisen), *Eubacterium* (Eubac), *Lachnospiraceae* (Lachno) and *Desulfotomaculum* (Desulfo). Ppcl23, Ppcl20, Ppcl18, Ppcl17, Ppcl24, Ppcl30, Ppcl21, Ppcl19 and Ppcl25 along with a sequence from *Protochlamydia* (Proto) formed Cluster 5 which was the cluster with the largest number of *P. penetrans* sequences. Ppcl1 clustered (Cluster 6) with four *P. ramosa* sequences Pcl4, Pcl3, Pcl35, Pcl5), two *Clostridium* sequences (Clos1, Clos5) and one sequence each from *Bacillus* spp. (B.LL01), *Paenibacillus* (Paeni3) and *Pithovirus* (Pitho). Ppcl33 most uniquely clustered, in Cluster 7, with all the seven Megavirus sequences that were used in the study along with two sequences from *P. ramosa* (Pcl1, Pcl2), one sequence from *Clostridium* spp. (Clos11) and one *Ruminococcus* sequence (Rumino2). Members of Cluster 7 were unique because they all had Lysine (K) and Aspartic acid (D) as the most occurring amino acid after Glycine. Ppcl8, Ppcl9 and Ppcl16 were separately clustered as Cluster 9. Most of the *P. ramosa* sequences (25 out of 37) clustered together in Cluster 1 with one *Clostridium* sequence (Clos3). Cluster 2 consisted of eleven *Bacillus* sequences (Bc2, B.JH7, Bt3, B.wied, Bt2, Bc6, Bc3, Bc5, B.weihen1, B.weihen2, Bc1) with one sequence each of *Clostridium* spp. (Clos2) and *P. ramosa* (Pcl29). Cluster 8 consisted of eight, three and one sequences of *Bacillus* spp., *Paenibacillus* spp. and *Sediminibacillus* spp. (B.amylo1, B.velez, B.safensis1, B.amylo2, B.pumilus1, B.pumilus2, Bc.ExsJ, B.acidicola, Paeni4, Paeni2, Paeni1, Sedimini). See File III, Table I (Supporting Information) for the codes used for the CLP sequences for different organisms.

## DISCUSSION

### The diversity of CLPs in *P. penetrans* Res148

The adhesive role of CLPs in the *Pasteuria*-nematode interaction has been hypothesised for a long time (Davies and Danks 1993; Mohan, Fould and Davies 2001; Davies and Opperman 2006; Davies 2009). This hypothesis was built on the knowledge that the endospores of many Gram-positive bacteria are covered with a hair-like nap, the fibers of which are constructed of glycoproteins containing G-X-Y repeat sequences, the number of G-X-Y repeats contributing to the length of the fibers (Sylvestre *et al*. 2003; Davies and Opperman 2006). While a diverse set of CLPs in the cladoceran parasite *P. ramosa* have been identified and characterised, the CLPs of *P. penetrans* and other nematode parasitic *Pasteuria* species remain unexplored. Here, we identify putative genes coding for CLPs in the genome of a highly host-selected isolate of *P. penetrans* (designated Res148). From a set of unpublished contigs, we predict 17 unique collagen-like sequences of which four putative CLPs (Ppcl23, Ppcl25, Ppcl26 and Ppcl33) were shown to have statistically significant similarities with 52 CLPs sequences from 22 different bacterial species and two groups of viruses. A cluster analysis of these sequences along with selected previously characterised CL sequences from *P. ramosa* and the BclA and ExsJ protein sequences from *B. anthracis* and *B. cereus* suggested that the CLPs in *P. penetrans* are extensively diverse. Since low complexity regions in proteins are more prone to non-erroneous replication slippage (DePristo, Zilversmit and Hartl 2006; Radó-Trilla and Albà 2012; Zilversmit *et al*. 2010) and are thereby susceptible to rapid evolution, these results suggest that the Ppcl sequences and their predicted homologs are evolutionary linked and they possibly evolved to serve similar biological functions. When compared with the phylogenetic diversity analyses using universal 16S rRNA gene, the cluster analysis of the G-X-Y repeat regions of CLPs suggest the latter to be an important source of variation and diversity on which evolution can act amongst *Pasteuria* spp. and other closely related pathogenic Firmicutes.

### Incongruencies in the phylogenetic resolution *Pasteuria* spp.

Preliminary studies had shown slight incongruences in the phylogenies estimated from different genes *gyrB*, *groEL*, *spo0A* and there was an observed phylogenetic biasedness between the trees based on the nucleic acid and amino acid sequences. This is likely because proteins are under different selective constraints due to their functional roles and are likely to be conserved over geological timescales (Huynen and Bork 1998; Romero and Arnold 2009). Biasedness in the phylogenetic resolution of *Pasteuria* spp. using CLPs suggests a real biological phenomenon more than just an artefact. One such known phenomenon could be due to incomplete lineage sorting, where a specific gene phylogeny is not congruent with the species phylogeny due to the evolutionary time-based selection pressures on different parts of a given genome (Degnan and Rosenberg 2006; Maddison and Knowles 2006). It is quite possible that this selection pressure did not allow the convergence of ancestries of individual genes of *Pasteuria* spp. to their overall observed phylogeny. Another phenomenon known to contributed to phylogenetic tree discordance is HGT which is known to facilitate bacterial diversity and plays a major role in bacterial evolution (Dutta and Pan 2002; Philippe and Douady 2003; Maddison and Knowles 2006). HGT events are also known to be an important source of ecological variances between closely related taxa (Cohan and Koeppel 2008; Wiedenbeck and Cohan 2011).

Since the nematode parasitic bacteria form a shared habitat with other soil-inhabiting bacteria, and HGT events between different species of soil bacteria is well-documented (Andrews *et al*. 2018), we suggest that these soil bacteria share a common gene pool, and therefore, they may have obtained these collagen-like sequences through HGT.

Interestingly, our results show an unanticipated sequence similarity of Ppcl33 with CLPs from Megaviridae. The members of the Megaviridae family are giant viruses (0.7 µm) that are known to infect protozoans in aquatic ecosystems. They have relatively large genomes (≥1.2 Mbp) which are predicted to encode more than a thousand protein coding genes including metabolic genes not found in any other viruses (Arslan *et al*. 2011; Colson *et al*. 2012; Legendre *et al*. 2012); they also possess hair-like structures on their capsids that are thought to be involved in adhesion and infection of their hosts. It has been postulated that Megaviruses acquired a large set of genes from bacteria during the course of their evolution through HGT (Filée, Siguier and Chandler 2007). The fact that *P. ramosa* is a bacterial endosymbiont of water fleas implies that both *P. ramosa* and Megaviruses possibly share the same ecological niche i.e. aquatic ecosystem and might support the view that HGT had occurred. However, our analysis did not identify any putative Megavirus CLPs in *P. ramosa* as might be expected. The CLP Ppcl33 was dissimilar to any other of the bacterial CLPs which suggests a totally different phylogenetic origin. In total, our analysis revealed five clusters of CLPs which would, therefore, suggest that the CLPs in *Pasteuria* are certainly not of a monophyletic origin; they therefore could have arisen *de novo* through mutation and subsequent selection, or more speculatively through selection acting upon G-X-Y repeats that had been acquired through several historic HGT events.

## Supplementary Material

Supplement Files
