# Supplementary material for: Evidence for diversifying selection of genetic regions of encoding putative collagen-like host-adhesive fibers in Pasteuria penetrans
Source: FEMS Microbiol Ecol. 2018 Oct 30;95(1):fiy217. doi: 10.1093/femsec/fiy217 (PMC6238073; doi:10.1093/femsec/fiy217)
Supplement: Supplement Files [file fiy217_supplement_files.zip › Supplementary File I.docx]

**Table I: List of accession numbers of 16S rRNAsequences used for phylogenetic analysis**

| **Sequence codes used in the phylogenetic tree** | **Accesion number**  **(database in parenthesis)** | **Organism** |
| --- | --- | --- |
| P.gottin | AF515699.1 (ENA) | *Pasteuria goettingianae* |
| P.hart | AJ878853.1 (ENA) | *Pasteuria hartismeri* |
| P.nishi | AF134868.2 (ENA) | *Pasteuria nishizawae* |
| P. ramosa | U34688.1.1435 (ENA) | *Pasteuria ramosa* |
| P.pen.HcP | JN592479.1 (ENA) | *Pasteuria penetrans* HcP |
| Ba.Ames | Ba16SA (KEGG) | *Bacillus anthracis* Ames |
| BA.AmesAnc | GBAA_5958 (KEGG) | *Bacillus anthracis* Ames Ancestor |
| BA.CDC684 | BAMEG_0007 (KEGG) | *Bacillus anthracis* CDC 684 |
| BA.A0248 | BAA_0007 (KEGG) | *Bacillus anthracis* A0248 |
| BC.ATCC14579 | BC0007 (KEGG) | *Bacillus cereus* ATCC 14579 |
| BC.ATCC10987 | BCE_5738 (KEGG) | *Bacillus cereus* ATCC 10987 |
| BC.AH187 | BCAH187_A0007 (KEGG) | *Bacillus cereus* AH187 |
| BC.G9842 | bcg:BCG9842_B5313 (KEGG) | *Bacillus cereus* G9842 |
| BT.AlHakam | (BALH_r16S01) (KEGG) | *Bacillus thuringiensis* Al Hakam |
| BT.kurstakiHD1 | BTK_r29390 (KEGG) | *Bacillus thuringiensis* serovar kurstaki HD-1 |
| BT.MC28 | MC28_r03 (KEGG) | *Bacillus thuringiensis* MC28 |
| BT.IS5056 | H175_rrna01 (KEGG) | *Bacillus thuringiensis* serovar thuringiensis IS5056 |
| Bs.168 | BSU_rRNA_1 (KEGG) | *Bacillus subtilis* subsp. subtilis 168 |
| Bs.spiziW23 | BSUW23_r20632 (KEGG) | *Bacillus subtilis* subsp. spizizenii W23 |
| Bs.natto.EST195 | BSNT_06293 (KEGG) | *Bacillus subtilis* subsp. natto BEST195 |
| Bs.BSn5 | BSn5_r21080 (KEGG) | *Bacillus subtilis* BSn5 |
| Paeni.poly.E681 | PPE_00010 (KEGG) | *Paenibacillus polymyxa* E681 |
| Paeni.muci.K02 | B2K_r38186 (KEGG) | *Paenibacillus mucilaginosus* K02 |
| Paeni.ter | HPL003_r28210 (KEGG) | *Paenibacillus terrae* |
| Paeni.larv | ERIC2_c00090 (KEGG) | *Paenibacillus larvae* |
| Pelo.UF01 | UFO1_R0122 (KEGG) | *Pelosinus* sp. UFO1 |
| Pelo.ferm | JBW_RNA0104 (KEGG) | *Pelosinus fermentans* |
| Clos.acet | CACET_c00110 (KEGG) | *Clostridium aceticum* |
| Clos.botu | CBOr001 (KEGG) | *Clostridium botulinum* A ATCC 3502 |
| Clos.tetE88 | CTC_0r03 (KEGG) | *Clostridium tetani* E88 |
| Micro.ela | AB001724.1 (GenBank) | *Microcystis elabens* NIES42 |
| Arthr.pla | AB074508.1 (GenBank) | *Arthrospira platensis* IAM M-135 |
| Gloe.HSC34 | EF150783.1 (GenBank) | *Gloeothece* sp. HSC34 |

**Table II:** **List of CLP sequences used for comparative studies with Pasteuria Ppcl sequences**

| Protein | Organism | Accession Number |  |
| --- | --- | --- | --- |
| Pcl18 | *P.ramosa* | ADU04102.1 |  |
| Pcl38 | *P.ramosa* | ADU04122.1 |  |
| Pcl37 | *P.ramosa* | ADU04121.1 |  |
| Pcl36 | *P.ramosa* | ADU04120.1 |  |
| Pcl35 | *P.ramosa* | ADU04119.1 |  |
| Pcl34 | *P.ramosa* | ADU04118.1 |  |
| Pcl33 | *P.ramosa* | ADU04117.1 |  |
| Pcl32 | *P.ramosa* | ADU04116.1 |  |
| Pcl31 | *P.ramosa* | ADU04115.1 |  |
| Pcl30 | *P.ramosa* | ADU04114.1 |  |
| Pcl29 | *P.ramosa* | ADU04113.1 |  |
| Pcl28 | *P.ramosa* | ADU04112.1 |  |
| Pcl27 | *P.ramosa* | ADU04111.1 |  |
| Pcl26 | *P.ramosa* | ADU04110.1 |  |
| Pcl25 | *P.ramosa* | ADU04109.1 |  |
| Pcl24 | *P.ramosa* | ADU04108.1 |  |
| Pcl23 | *P.ramosa* | ADU04107.1 |  |
| Pcl22 | *P.ramosa* | ADU04106.1 |  |
| Pcl21 | *P.ramosa* | ADU04105.1 |  |
| Pcl20 | *P.ramosa* | ADU04104.1 |  |
| Pcl19 | *P.ramosa* | ADU04103.1 |  |
| Pcl17 | *P.ramosa* | ADU04101.1 |  |
| Pcl16 | *P.ramosa* | ADU04100.1 |  |
| Pcl15 | *P.ramosa* | ADU04099.1 |  |
| Pcl14 | *P.ramosa* | ADU04098.1 |  |
| Pcl13 | *P.ramosa* | ADU04097.1 |  |
| Pcl12 | *P.ramosa* | ADU04096.1 |  |
| Pcl11 | *P.ramosa* | ADU04095.1 |  |
| Pcl10 | *P.ramosa* | ADU04094.1 |  |
| Pcl8 | *P.ramosa* | ADU04092.1 |  |
| Pcl7 | *P.ramosa* | ADU04091.1 |  |
| Pcl6 | *P.ramosa* | ADU04090.1 |  |
| Pcl5 | *P.ramosa* | ADU04089.1 |  |
| Pcl4 | *P.ramosa* | ADU04088.1 |  |
| Pcl3 | *P.ramosa* | ADU04087.1 |  |
| Pcl2 | *P.ramosa* | ADU04086.1 |  |
| Pcl1 | *P.ramosa* | ADU04085.1 |  |
| Proto | *Protochlamydia naegleriophila* | WP_059059638.1 |  |
| Eisen | *Eisenbergiella tayi* | WP_069429780.1 |  |
| Rumino1 | *Ruminococcus torques* | WP_070103488.1 |  |
| Eubac | *Eubacterium dolichum* | WP_004799529.1 |  |
| Fusica | *Fusicatenibacter* sp. | CUQ46188.1 |  |
| Desulfo | *Desulfotomaculum guttoideum* | SEU24404.1 |  |
| Lachno | *Lachnospiraceae* bacterium.mt14 | WP_053983733.1 |  |
| Rumino2 | *Ruminococcus* sp. JC304 | WP_019163773.1 |  |
| Ba.BclA | *B.anthracis* | WP_000069710.1 |  |
| Bt1 | *B.thuringiensis* IBL200 | EEM98224.1 |  |
| Bt2 | *B.thuringiensis* serovar israelensis | EAO54405.1 |  |
| Bt3 | *B.thuringiensis* serovar huazhongensis | WP_001288630.1 |  |
| Bt4 | *B.thuringiensis* serovar Berliner | WP_003271363.1 |  |
| Bc1 | *B.cereus* | WP_050567719.1 |  |
| Bc2 | *B.cereus* | WP_059303846.1 |  |
| Bc3 | *B.cereus* AH1273 | WP_002079900.1 |  |
| Bc4 | *B.cereus* AH603 | EEL67218.1 |  |
| Bc5 | *B.cereus* AH1272 | EEL88100.1 |  |
| Bc6 | *B.cereus* AH1273 | WP_002079900.1 |  |
| Bc.ExsJ | *B.cereus* ATCC10876 | AAN85822.1 |  |
| B.acidicola | *B.acidicola* | WP_066270930.1 |  |
| B.LL01 | *Bacillus* sp. LL01 | WP_047972494.1 |  |
| B.JH7 | *Bacillus* sp. JH7 | WP_061139390.1 |  |
| B.pumilus1 | *B.pumilus* | WP_060597094.1 |  |
| B.pumilus2 | *B.pumilus* | WP_045209866.1 |  |
| B.safensis1 | *B.safensis* | WP_075612411.1 |  |
| B.wied | *B.wiedmannii* | WP_064459749.1 |  |
| B.velez | *B.velezensis* | WP_015240391.1 |  |
| B.weihen1 | *B.weihenstephanensis.*KBAB4 | WP_012261565.1 |  |
| B.weihen2 | *B.weihenstephanensis* | WP_038626174.1 |  |
| B.amylo1 | *B.amyloliquefaciens* | KJD59259.1 |  |
| B.amylo2 | *B.amyloliquefaciens.*EBL11 | WP_032866501.1 |  |
| Clos1 | *Clostridium arbusti* | WP_010235513.1 |  |
| Clos2 | *Clostridium formicaceticum* | WP_070965690.1 |  |
| Clos3 | *Clostridium botulinum* | WP_052705854.1 |  |
| Clos4 | *Clostridium beijerinckii* ATCC 35702 | WP_012059564.1 |  |
| Clos5 | *Clostridium tyrobutyricum* | WP_023625409.1 |  |
| Clos6 | *Clostridium pasteurianum* NRRL | ALB45444.1 |  |
| Clos7 | *Clostridium diolis* | WP_039769245.1 |  |
| Clos8 | *Clostridium formicaceticum* | WP_070968717.1 |  |
| Clos9 | *Clostridium paraputrificum* | WP_027098858.1 |  |
| Clos10 | Clostridiales bacterium.VE202-06 | WP_049923917.1 |  |
| Clos11 | *Clostridium botulinum* | WP_061312809.1 |  |
| Clos12 | *Clostridium* sp. CAG:265 | CDB74255.1 |  |
| Sedimini | *Sediminibacillus albus* | SDK36124.1 |  |
| Paeni1 | *Paenibacillus swuensis* | WP_068611280.1 |  |
| Paeni2 | *Paenibacillus pabuli* | WP_068964107.1 |  |
| Paeni3 | *Paenibacillus mucilaginosus* | WP_063634302.1 |  |
| Paeni4 | *Paenibacillus* sp. ov031 | WP_072735197.1 |  |
| Pitho | *Pithovirus sibericum* | YP_009001250.1 |  |
| Mega1 | *Megavirus lBa* | AGD92195.1 |  |
| Mega2 | *Megavirus chiliensis* | YP_004894340.1 |  |
| Mega3 | *Megavirus courdo7* | AEX61385.1 |  |
| Mega4 | *Megavirus courdo11* | AFX92324.1 |  |
| Mega5 | *Megavirus lBa* | AGD92520.1 |  |
| Mega6 | *Megavirus chiliensis* | YP_004894639.1 |  |
| Mega7 | *Megavirus courdo11* | AFX92662.1 |  |
| Mega8 | *Megavirus courdo7* | AEX61765.1 |  |
