# Supplementary material for: Evidence for diversifying selection of genetic regions of encoding putative collagen-like host-adhesive fibers in Pasteuria penetrans
Source: FEMS Microbiol Ecol. 2018 Oct 30;95(1):fiy217. doi: 10.1093/femsec/fiy217 (PMC6238073; doi:10.1093/femsec/fiy217)
Supplement: Supplement Files [file fiy217_supplement_files.zip › Supplementary File II.docx]

**R-Script for Heatmap**

################ Installing and loading required packages ################

if (!require("gplots")) {

install.packages("gplots", dependencies = TRUE)

library(gplots) }

if (!require("RColorBrewer")) {

install.packages("RColorBrewer", dependencies = TRUE)

library(RColorBrewer) }

############# Reading in data and transform it into matrix format #############

data <- read.csv("GXYpercentcompositionmatrix.csv", comment.char="#")

rnames <- data[,1] # assign labels in column 1 to "rnames"

mat_data <- data.matrix(data[,2:ncol(data)]) # transform column 2-5 into a matrix

rownames(mat_data) <- rnames # assign row names

rownames(mat_data)

data

mat_data

############# Customizing the data and parameters for heatmap #############

# creates a own color palette from red to green

my_palette <- colorRampPalette(c("white", "red"))(n = 299)

# (optional) defines the color breaks manually for a "skewed" color transition

col_breaks = c(seq(-1,0,length=100), seq(0,0.8,length=100)) # for red and yellow and green respectively

distance = dist(mat_data, method = "manhattan")

cluster = hclust(distance, method = "ward.D")

cc<-sapply(1:108, function(i) ifelse(substr(row.names(mat_data)[i], 1, 1)=="B", "skyblue", ifelse(substr(row.names(mat_data)[i], 1, 4)=="Clos", "blue",ifelse(substr(row.names(mat_data)[i], 1, 5)=="Paeni", "orange", ifelse(substr(row.names(mat_data)[i], 1, 6)=="Viridi", "orange",ifelse(substr(row.names(mat_data)[i], 1, 4)=="Sedi", "orange",ifelse(substr(row.names(mat_data)[i], 1, 5)=="Pitho", "green4", ifelse(substr(row.names(mat_data)[i], 1, 4)=="Mega", "green",ifelse(substr(row.names(mat_data)[i], 1, 4)=="Ppcl", "purple", ifelse(substr(row.names(mat_data)[i], 1, 3)=="Pcl", "pink", "black"))))))))))

########################## Heatmap generation ##########################

heatmap <- heatmap.2(mat_data, density.info="none", # turns off density plot inside color legend

trace="none", # turns off trace lines inside the heat map

margins =c(8,5), # widens margins around plot

col=my_palette, # use on color palette defined earlier

dendrogram="row", # only draw a row dendrogram

Colv="NA", # turn off column clustering

RowSideColors = cc,

keysize=1.0, key.par = list(cex=0.5))

install.packages("ape")

library(ape)

tree <- as.phylo(cluster)

write.tree(phy=tree, file = 'GXYcomposition.tree')
