# Supplementary material for: Evidence for diversifying selection of genetic regions of encoding putative collagen-like host-adhesive fibers in Pasteuria penetrans
Source: FEMS Microbiol Ecol. 2018 Oct 30;95(1):fiy217. doi: 10.1093/femsec/fiy217 (PMC6238073; doi:10.1093/femsec/fiy217)
Supplement: Supplement Files [file fiy217_supplement_files.zip › Supplementary File III.docx]

Nucleic acid sequences of 23 putative collagen-like proteins in *P.penetrans* Res148

>Ppcl1

ATGTCCAACCTTGAATTACTACACCGCCTATGCTGTTCATGCTGCCCAACAGCACCAACAGGTCCAACGGGACCAACATGTGTGTGTCCTCCGGGACCGCCAGGACCAGTGGGCCCACAAGGACCACAGGGACCGGCAGGACCACAGGGGCCACCAGGACCGGCGGGATCGACATGTGTATGCCCCCCAGGTCCAACAGGACCACCGGGGCCACAAGGACCAGCGGGAACACCAGGAGCACAAGGACCACAGGGACCACAAGGACCACAGGGACCACAAGGGGCACAGGGATTACAGGGACCGGCAGGAACACCGGGAGCAGCGGGACCGGCAGGAACACCGGGAGCAGCGGGACCGGCAGGAACACCGGGAGCAGCGGGACCGGCAGGAACACCGGGAGCAGCGGGACCAGCAGGACCAGCAGGGACACCAGGAGCACCGGGGACACCAGGAACACCGGGAGCAGCGGGACCGGCGGGAGCAGCAGGGGCAGCAGGTCCAGCAGGGGCAGCAGGAACACCGGGAGCAGCAGGTCCAGCAGGACCAGCAGGGGCAGCAGGTCCAGCAGGAGCACAAGGGTTACCAGGACCAGCAGGGGCAGCAGGACCAGCAGGGGCAGCAGGTCCAGCAGGAGCGGCAGGTCCAGCAGGAGCGGCAGGTCCAGCGGGACCAGCGGGAGCAGCAGGACCGGCAGGACCAGCAGGAGCGGCAGGACCGGCAGGACCAGCAGGAGCAACAGGACCACAAGGACCACAAGGACCACAGGGAATACAGGGGATACAAGGAATACAAGGAATACAAGGAATACAAGGAACAACCGGTGCCACAGGACCTTGCTGCGCGGTTAGCTCCGCATATTACCAAATAATATCATTCGATTCGACCCTCCCAATAACCAATATAATTTACGAAAATGGACCAAATATTAGGAGTTTTATTGGTTCAACTGTTATTTCTCTAGAACCAAACAGAATTTATGAGGTAATTGTTGGCTTTACCAATGGTAGTAATGGTGTTTTGGGTGTAATTCATCCTACCTTAAATGGTGTATTTCTACCCTTTAATGTGGGTACGCCTATTAACCAAGAATTGGAAAGATCCTTCCTTGTGCCAACCCCACCTGGGGGAAATAGTTTACTGAGTCTAGTAATTACGCGCAATCAGGGATTTTTGGCTGGACGCCAAGTAGTGGTAATAGAATTACCGAGTGGTAAT

>Ppcl8

ATGCCCAATCATTCGGGTTTAAGAGGTTCCCCTTTGGATCCGGAAATATCCCCTAAAAGGCCCATTCAGGAATTCACACCCATAGTAGGATTCTGCCGGTCCAGTTCTTGCGGCTCCACTGGTCTTGTTGGTTTCTGTGTTTCCACTGATTCCTGTGGTTTTATAGGTCCCTCCGTTTTTTGTATCCCTACTGGTCCTTCGGGTCTCTGTGTTTCTGGTGTTCCTACCGATTTTGGTGGTCCCATGGATCCTGCTGCTGGTCCCTGTGCCCTCGGCATCTTTGCCGATCCCAGTGGTCCCACTGATTTTGCTGGGCTCTGTGTTCCTAATGTTTTCACTGTTCTTGTTAGTCCTGTAGGACCTGTTAGTCCTGTAGGACTTGTTGGTCCTGTGGGACTCGTTGGACTTGTTGGCCCCGTAGGACTTGTTGGACTTGTTGGTCCTGTGGGACTCGTTGGACTTGTTGGTCCCGTAGGACTTGTAGGCCCTGTAGGCCCTGTAGGCCCTGTAGGACTTGTAGGACTTGTAGGACTTGTAGGCCCTGTAGGACTTGTAGGACTTGTAGGCCCTGTAGGACTTGTTGGTCCTGTTGGTCCTGTTGGCCCCGTTGGTCTTGTTGGTCCCGTCGGACATATTATTTTTATTGGTTTTGTTGGTTTTACCGGACCTGTTGATCCTCTCGAACTTGTTGTCTTTTTTGGACCTGTTGGTTTTGGCGGATTTGTTGGTTTGGTAGAAAATCGTGGTGGATTA

>Ppcl9

ATGATATCGGTAGTGGTAACAATGACATCACCACTTCCAAATATTGTCCCGCTAGGGGTTACTAGGGTAATCGGTGTTGTAGTATTCGGAGCAAGTGTTTGGATGTCTCCAGCAGTGGAGGGAATTACGGTAACGTCTGTTGTTATGACTGGACCGGTTGGCCCTGTAACCCCCGTAACCCCTGTAATCCCTGTAATCCCCGTAATCCCTGTAATCCCCGTGGGTCCCGTGGATCCTGTAGCCCCTGGATCACCCGTAGGTCCCGTGGGTCCCGTAGGTCCTGTGAATCCTGTAAATCCCGTGGGTCCTGTGGGTCCCGTGGGTCCCGTAGGTCCTGTGGATCCCGTGGGTCCCGTAGGTCCTGTAAATCCTGTGGGCCCTGTAATGATGCTGATATTACATATGGAGGGGAAACCGCAAGGGGGAAATAAATTTAAAATCATGTATATTTATCCCTTCTTAAATAAATTCTTATTGTTTTATTTATTTTTTTTAAAAGTAAATTCAGATAAAATAACATTTTTTTATGATGACGATTGCCGGGAATCATTCAAGAAGAGGATTTCCTGGGGATTTGGGTCCCTTGGCCGAAATCTGGATCCAAGGGATTACCCCATGTTCCCCCCTCGGACATCAAGGTCCCCCCACGCAGAAATGGACTACCTACCA

>Ppcl16

ATGTATCATAATGATTACCAGGGAAAAATGAGTGATTATTATAAAGAATACTATGATTTCTATTATGATTACTACAAAAATATAGAAAATAACGATTGTAAACACAGAGGGGATAAAAATTATCATAATTACCATCAGCAACAACCCCACCATGAGCAGCAATGTTGTCCGCCTCCTCAGGAGTGCTGCCCCATTGTAGGACCACCAGGACCACCAGGACCACCAGGATCACGGGGTTGCCCAGGACCACAGGGTTCCCCAGGACCACAGGGCTGCCCAGGAAAACAGGGACCGCCAGGACCACAGGGTTGCCCAGGACCACAGGGTTGCCCAGGAAAACAGGGACCACCAGGACCACAGGGCTGCCCAGGAAAACAGGGACCACCAGGACCACAGGGTTGCCCAGGACCACAGGGACCACCAGGACCACCAGGACCACCAGGACCACCAGCCCCACCATGCCCTCCACCATGCCCTCCACCATGCCCTCCACCTCCTCCACCATGCCCCCCACCATGTCCTCCACCTCCCCCACCATGTCCTCCGCCATGTCCTCCACCATGTCCTCCACCATGCCCTCCACCACCATATCCCCACAGGGAATAC

>Ppcl17

ATGAAGCGTTCAACAAAATATCCATTCTTGGCTATGCTTGCATTAGTATCCACTGTGCGGGCGCAAGATTGCCCTTCATTTGCGGTGGTAACACCCCAATGCGCAACAGCACTTAATACATTACAGGCTCTCATATCGCCTGCACAGGCTGGCGCACCAATAACAGGCGATACATACATGAAAGTGGAAGATGCACATGCCCTTATTAAAGAGGCGTTTAATGTACTGAACATCATAGAGAGTGATCAAAGCAACTACCAAAAAATTGCACAATCATTGCATGAATATCAAAATGATCTTGATAATGGCTTACACGTAGTTGATGCAGGCTACGTGTCAACGCGTAATTGCAAAGTTGTTGACTCTGTGTGTGTGAAAAACTCGTTGGGTGTAGGCGGCAATGCACAGGTTAGAGGTCAAGTATCAGCAGGTAATGTTGTTGCATCTAATGTAGTAACAAGCTCAGCAACGGCTGATTTGGTTAGCGCCTTTTTATTAGATGGGCAGATTATTAATGCCTCAAGTATTTTTGTATCTGAAATAAATGGCATACCGCTTAGTGCAAGTGGCCTAAATATTTTTGGTACGCGGGGAGCAACCGGTCCGCAAGGAACTACAGGAAACACCGGACCAGACGGTAACACCGGTCCTGGCTCTACCGGGGCAGCAGGAGCTGTTGGTTTGACAGGCAATACGGGCATACAAGGTATTACCGGCAATACCGGTCCAGTGGGTATAGTCGGTAGCACCGGACCAGATGGTGGTTTTTCTGGTGCACCGGCAGGGTACCTTAATAGATTTAATAACGTGAGTGCGCCGGTGGGTAGTCCAGGTGGTTTTGCTCCCATAACGTTTGCATCAAGTGCTCTTGCAGAAAATGGTTGGACTACAGTAAACAATATTACGTTTACCTGTCAAATTGCAGGGCTTTATTTAATTTCGTTCTCAGGTAATTTTTTATTGCCTTTAGTTGATCAATTTGCACGTATAGGAATGAGAATTGTGGTGAGCGTGGGTGATAATCAGGCATATTATGGCAGGGTAAATGAAACGTCACCAATTGCCGGCGGTATAAGTACTTTTTTGACAAACTCGGCATTAGTCAATTGCATTGTTGGCACAACGGTGCGTGTTGAAATGACCGCCAGTGTAGCAGATGTTATACTATTTAATCCTGCATTACAGTCTACGGTAAATACACAGGGCCAAGCCGCTAATTTAATAATCAGACGAGTATTTTAA

>Ppcl18

ATGAAGATTAAAACACTATTACTTTTTATATTAGGTAGTATTTCATTCCACTTGTCCGCTCAGGAAGAGCATTTACAAAAGATATGCGTTGATAAGGAGTTAATAGACGCGTTAAAAACAATTAAATCAACGTGCCGTGGAATAATTTCTCCTGCAATTTTTGCGACGTGTGACGCATACGATTATGGGTATACCCAGCTGCCCTCATGCATGGTAAGTTCTGCAATAGATGATGCCTTATTTTATGTAGAACAGTTGAAAAATGACCAAGCGCAATTGCTTAAAACGTTTTTTCAAAGCTATCAATTGTTGTTGCAGGATCGATCCTCAAAGAAAAATAAAGTAATTAATAATTTAATTGTTCAGGAATCGGCTAAAGTTAAAAACTTAGTTGTGGGCGGATCAATTATAGGTTCTTTTGCCGGTACTAATTCGGTGGTTAATGGCGTTACGGGCTTAACGGGTTTTACCGGTAATACCGGGTCTCAACTTCTTGCACGTGGAGCTACCGGAAGCTCGGGAGAAACGGGTAACACCGGAGCTACCGGTTCTACCGGCGTTACCGGCTTAATTGGAAACATTGGGGCTGCTGGTGCATTAGGCGCTACTGGTCCTACTGGCGTAACGGGTTTAACCGGTTTTACTGGTCCACGCGGTGATGCAGGATTGCAAGGCCCAAGCGGTAATACCGGTGCACCTGGTGTAAGACTCCCTTTTGCCTACAGATTCAATACTACTAATCCCCTAACATTTACTCGTGGTCAGGTGTTATCTATGCCGAATGTTATATTCTTTGGTATGACCGCGTCAACGCCTGATACCGTTATTTTTAACACCAGTGGGGTATTTGAAGTGTTTTATATTATTAATGGCTTTCGTGCTACAGCTGGTAATGTGCCTCTATCACCCGAAGTTCAAATTTATGCAATTAATGCCGCGGGTAATATTATAGATGGTAGTACCTACGGGTACCGGATTTTAAATGCATTTCCGGTTGATTTTGGTCAACAGTCTATATGTGGACAGTTTATTATGCAAGCTAATGCAGGCGATTCCGTACGATTAGTTAATAATACCGGTGCTCCTACCAATTTGTTAAACGTGAATGCTCAGGCTGGTAATGCTGCAACCACTACGAGTATTAGCATGTATGTGAGACAAATTGCATAA

>Ppcl19

ATGATAATGAAAGCTATTTTAAATATATACCTGATTTTTGGTTACGTATTTTGTTTTCAGCTCATCGCAATGCATGCAACTGAACAAATTCTCGTACCTGAATATGTAAAACATGTACTTTTGGACATCAAAGATAACAATTGCATACGCGTGTATGATAATGCATTTGATGAATTGTGCACAGCTCTTAAATCAAGTGACAATGTTGATATGAGTGTGATAGTAGCCGGAATTAATCATTTACTTGCGTTTGAAAATTCATTACCAGAATATTCACAAGAACTACATAGTTTAAAGCACTACAAAGAGTGTATTTCTACTGATGAACAGAGTTGTGTAGTGAGTAAATCAAAATGCAAAGAGTATTGCAGACTCTGCGCAGAAACATTAAAAGTTGCCGGTAACTTATGCGTAGGGGGATTAATCTGTGCACCCGAAGTGTCTGATCCTTCCACTGTTGGTCTAGACGGGGCTCGTGGACCTCAAGGTAACACAGGAGCACTTGGCCAGACAGGAGATACGGGTATTACCGGTTTAACCGGATTAACTGGTCTTATAGGTGCTTTAGGAGCACAAGGAGCGCAGGGCCAAACGGGAGATACGGGTTTTACCGGATTAACTGGTTTTACCGGTCCCGTAGGCAATGCCGGTAATCCTGGCATTGCAGGACCACTGGGAGCTGCCAATCTGCAAACAGGGATCACCGGGCCAACTGGCGCAAGCGTAACCCCTCAAGCATATGCAATGTTTTTGGTAACCGGTGTAAGTGGGTTAGTAGTGCCTATTAACACGGGAATTAATTTTGCAGGATCTATACCTACCGTACCAGTAGGCTTGTCCCTAAATGGTGATACTATTACTATTGCCGAACCAGGAACGTATGAAATTACATATATTGTAACTGAAGGCGGTGGCTCTGGTTTACAATTAGGATTGTTAGTAAATGGTGTTGTAGATCCAAATTTTAATCGAGTTTCAAATAATCCTTATTCGCAAATGTATGGTCAGGGATTGTTAAGTGTTACCCAGCCCAATACTCAAATTGTTTTGTTTAATTTTTTTCTACTGACCTTAAATGGTAATTTGGGCGGCGATAATTTGGGTACTGCAGCTTCACTACTTATTAAACGGATTGCAAGTTAA

>Ppcl20

GTGCGTGGAAATGCGCGCATTGGCGGTAATTTAATTGTGTGCGGAACCATATGTCCTGATCCACGCACTGGTCAAGCGGCTGCGGGAGACCCAGGTGCTACGGGTGCTACGGGAAGCACCGGCTTTACCGGATTTACCGGACCGCAGGGTGCAGTTGGAGCTCTTGGTGCTGCTGGACAAACAGGTAATACCGGCCCAACCGGCTCTACTGGATTTACCGGACCGATTGGCACACAAGGTGCAGCAGGTGCACAAGGTGAAACAGGCAATACTGGTTCTACAGGGCTTACTGGTTTTACGGGGCTTACGGGCAATGTGGGACCTGCGGGTACACCTGCAAATGAATCACTGTATGCATCGTATTATTTTACCGGTGCCACGAGCGTAACCGGTGGCGCAGCCACAGATGCAGTGGGAGCGGCATCTGTTCCATTTACCACTCAGGGACCTGTAAATGGTTTTAACCTGATAGGTGGCACTGATATTCAGGTAACTACAACAGGTATATATGAATTGACCTTTCAAGTTTTAACATTCCAAGAAGCAAATCTTTTTGCTGTTACGGTAAATGGTGTCATTGTTACCCGTTATACATCTGGTCAAAAGACTTCATCGCCATATGGTTTAGGACGTATTCTAGTAAATGCCAATGCAGGAGATATTATTAATATTAAAAATATAGGTGTAGCCTTATCGCCTTCGGTTGTTGTTTCAGTACCGTTGTCGTATGGATCTGGAGATCGGGCTACGGCTTCTGTAATGATCCGGCAAATTTTTTAA

>Ppcl21

ATGTTAGAGTTTCACCTTCCTGAGTCGAGCTATATAAAAGACACGGTTGCTATTAGTGATGAGGCTAATCTGGTGATTGGATCTTTTGCCACACCAGAGCATGAATCTTGTGCATTATGCTTTGATTTACACGGTAATTTTTTATCCTACATTGTCGCTCAAGACAAGGTAACAAAAAAAGCCTGCAAAATTTTTAACAATGTATGTGTTAGGAATAATGTAAAAATTTGTGGTGATTTACTTGTGTGTGGCCGCATCATCAATCCTGATTGTTTTATAAGAAGAAGTATTACTGAATGTAGCAATGGTATTACCGGAGCCACTGGTCCTCAAGGAAACACCGGACCTGCAGGTCTTAGTATTACGGGGGCCCAAGGAAACACCGGTCCTGCAGGAAATACGGGCAATACAGGACCAACTGGTCCACAAGGTCAAATTGGTCTACAGGGTAATACTGGCTCAGCAGGAGCTACTGGCATTCAAGGTGAACCGGGCAATACGGGCACTACAGGCCCAACGGGATTGCAAGGTTTACAAGGCACAACAGGAAATACTGGCCTACAAGGCATTCAAGGCAATACGGGTGCAACAGGTGCTACTGGCAACACAGGGTCTATCGGTGCGCAAGGTAGTACGGGGCCTGTTGGTGCTACTGGTTTTACCGGCGCTACAGGTTTTACTGGTCCACAAGGTCAAACGGGCAATACTGGCTTTACGGGATTAACGGGGGCAACAGGCGCTGTTGGTAACACTGGGTTTACTGGTCCGCAAGGGCAAACTGGGTTTACTGGCCCGCAAGGTGCTACTGGTTTTACCGGTCCAACTGGTTTTACCGGAGTGCAGGGCCCACAAGGCAACACTGGATTTACCGGAGCTCAGGGCAATCAGGGCTCAACGGGCTCAACGGGTTTTACCGGCCCAACTGGTTTTACGGGTGCTACCGGTATACAAGGCAATACAGGATTAACGGGTGTAACGGGCGCTCAGGGTATTCAAGGTAACACCGGTTTGCAAGGATCAACGGGTTTTACCGGAGCCACCGGTATTCAGGGTAGCACGGGTTTGCAGGGTTCAACGGGGTTTACCGGCGCTACAGGTTTTACTGGTCCAACAGGCTTTACGGGTGCTACGGGTAATACCGGATTTACTGGTCCTCAAGGTGCTGCTGGCGCTGTGGGAGCACAAGGTCCGCAAGGGACAACGGGTAACACTGGTCTGCAGGGTACTACTGGATTTACGGGCAGCACAGGGTATACAGGATTTACCGGCCCGCAAGGCCCTACCGGGGTAACAGGAGTTACCGGTCCACAAGGTGCTGTGGGATCTACTGGTTTTACCGGAGCCACCGGTGCACAAGGACAGACCGGTAATACCGGTCCGCAAGGAGCTACAGGCAACACAGGGCCACAAGGTGTTACTGGCAGTACCGGAAATACGGGCGAACGCGGCAATACCGGGCCTACGGGAACTGCTTTAGAACCGGCAAATTTTGTTAGCTCGTATAGCCTTATATCAGCAACTGCTCCGCAGAATGCGTATACTGGCATTCAATTTGATCAAAATGCAGTGCCGCCTGCTGGGTGGACAAAAACTGCCGACACATTTATTTGCAACCAAGCCGGAATTTATGAGATGTCCTATGTTATAACCGCGGCTGCCGTCACTGGATTTAATACCCAGCTAGTATATTCTCGTATTTTACAAAATGGTGTAACAGTAGTTCCCGGTAGTGTTATATCGGTATCATGGAATAGTACTAACACGGGAAATCTCACTAATATATTAGCGCAAACGGTTCTTGTTTCAGCTTCAGTAAATGATGTTTTTCAGCTTCAATTTGGTGCAACTAATGCGAATGCGATATTTGTACGCCCGATCACAACAGGTGGTTTAGGTACTTCATCGGGCGCTTCTTTTACTATTAGAAGGGTTGCGTAG

>Ppcl23

ATGCTTGCCGTTTTGTTGTCTGCGCCGCTGTGTGCACAGGAGTTTGATAATTGTGATGAACTATCTTTAGCTGAACAGCGATACATTGCATCAGTACAAGAAGAGGGCGTCTTTATACCAGAATTTATTTCCTATTCAATACAAACTGTTGCTAAGTTTTTTCCACGAGCCGTGTACAATGATTCATTTCTGGAGCTGCGTTGCCATGCAAAAGATAACGTGCGAATAATTGCATTATCAAAGGCTATACGCGCAGTTGATCAAGCGTTGCAACTTGCAGCTCAACGTGAAGAACTTGCTGACGTTGTTGCGTATTTAACAGCGTACAAATCGCAAATACTCTCAGGCCAAGCAATAATTTCTTGTGACAGAAAACGATTAAAAACCTATTGCCAAGCATGTGCTCGTATTTTAAAAGTATGTAACCTGACGGTTTGTGGTGCAATTTCAATTCCAGGACTATCAAGTTCATCCGGTTCATTAGGCGGCAATGGAGCAGCCTCGCTTGGTGATCCGGGTGCTACAGGGTTTACCGGTGCTACCGGCTTAACCGGACTAACTGGTTTGACAGGATTTACCGGAGCACAGGGTGCCGTGGGCGCACAAGGTGAACCCGGACAAACAGGCATAACCGGACAAACGGGCCCAACTGGTAGTGTGGGCAGTAGAGGTTTGCAAGGCCCAACAGGAGCGCAGGGTGACTCGGGTACTACTGGATTTACCGGACCTATTGGGTCTGCATTGGCCGATGACGCATATGGCTATTTTTATTACAACTCAAGTTCATCAGTTGGTAATGGAAGTCCTGTACCGCTCAACGTGCAAGGTACCGTAGTAGGGACCAGCTTGCAGGCCAATGGTGTGATAAATATTGTGCCAGCGGGTACCTACAAAATAAGTTTTGCAGTAGCAACTAATAATCCAAACAAATTTGGTTTGAGTATCAATGGTGCAGCGCCGGAAGCACGCACAATTTTTGCACAGGGTGCAAGTAACGCGATGAATTTTGGGGAAGTTATTCTCACGGTTGGTGCAAATACCAACGTGCAAGTTGTCAATGCAACGGGATCTTCAATTTTAGTATGGGGATTGTTGGGTGGAGCTGGTGCGCCGCCTAATAGTATTTCTGCTTCAGTACTAGTACGTAGAATTGCATAA

>Ppcl24

ATGAACGAAGTAACTCAACTCTCTCAAGCCGATTATGATTTAATTGCAGAAGCAGCGGATAAATCATTTGTGTATATTCCTGATGGTGTTAAAGCAGCATTGAGCGAAGCAAATACAGAAAAATCAATGTCATCCGACACATTACAACTCATGAAGTGGCTCGAGCAGGGCAATCACTTATATTCTATGTCTGATACCAAAAATATATTTAAAGAATATAAAGATATACCAGCTACCAATCAGCATGAGCTATGTCTTGTACATTTTGCACAATTGGTTGATCAAGAAGATTCGCTTGTAGTAATTGATGATAGCATCGATCGTAAAAAGCCAAAAGTTTACACCAATTTATTAGCCCACATTTTGCAAATTGGTGGCAACCTCATTGTTAATGGTCTTATCAATGTGCCATCAGCCGGAACAATCAGTGGTGGCGCAACAGGCGCTGATGGCTTTACTGGAGCAAAAGGTCCTACAGGCCCTATTGGCCAAACGGGTAATACTGGTCCTGATGGCTTTACAGGCTTTACTGGTTTTACCGGCAACACGGGTTTTCTTGGCAACACAGGTGTTACTGGTGCTACGGGACCAAATGGTTCAACTGGCTTTACCGGTTTTACTGGCTTTACTGGTAATATCGGAGCAACCGGATCAACAGGGTCCACGGGTGCTGTTGGAGATACTGGCCCGGTCGGGCTACGAGGTGCCCAAGGCCTTACTGGCAACACGGGACCATCTTCATCCATTACTGGTTCAACCGGCCAGACCGGTGCGTTACTGGGTGCGGCTGCAGCATATGGCTTTTTTTACACAACCGGTGCTGTAACGGGTGTTGCGGAAACGGTGACGCTATTATCAGCATTTACCTTTAGAGGTAGTATTCCAGCAGTCACTCCAGGTATTGCAAATGGGGGTAGTATTATCACCATTGCCGATGCAGGCACCTATAAATTTAACTATATTGTGCAAGGAGTGACCAGCAGTGTCATAGGGTTGCAGGTTAATTTTGTAACCGATCCAGTTACTATTTTTGCTCAATCATCTGCCAATTCACAAAACGTAGGTGAGGCAATTCTTACTGTACCAGCAGGCGCATCGATCCGGTTGATGAATTTGAATACTGCAAACCTTAATGTGCTTAATTCTTTGGGTGGCGATGTTCAAGGAACGGCATTTTCTTTAATGATTAGAAGATTGAACTAG

>Ppcl25

ATGAAAAAAATTATTATTTACTTATTACTTATCAGCAGCGTGGTGCAAAGCACCATAACTGCATCATTTCTGTTGCCCCATGATCTTGTCTCAGCTTTACAAGAAATAGTGCGCTTGAGTGGACAAGAGGTTTTGTCTCCCGCTTTGCAGTTATTGTGCTCGCATATTGAAGAACATGATTACGTAGTATCTGAAGATATAGTAGGCCATGCATTGCCCCAAGCATTACAGTATGTACAGGATCATAAAGATCAATTGCAATCAGATCGTTACCAAGCGGCTCTTGATGCCATTAATCAGACTATGTCTGATATAGAGCACAACAATGTAGTGACAGTCGATGATGCGACTACAAGAAAACATGTACACAATAAAGCGTTTGATAATCTTATTGTAAGAGGCAATGTTAAAGTAAGAGATCTTATTGTCTGTGGTGCACTAAACGGCACCGTTAATTGTGGCACGGGCATTGGCATAAACGGGCAAACAGGAATTTCTATCACTGGTGTTCAGGGTTTGCAAGGTGTAGCGGGCGCCACGGGTGATACCGGTGCTACAGGAGTAACGGGTGATACTGGATTGACGGGTGTTATTGGTCCGGTAGGTATTGCAGGAGCTCAAGGCGAGCAAGGCCAAACAGGTCCTACAGGAATTACTGGTCTGTCTGGTTTTACTGGTAACCAGGGTCCAACGGGGATTCCAGGAAGCACAGGTTTTACCGGTGAGCCGGGTGTGGCACTCAGTTATGCCTATATTTACAACACGGCGCCACAAACGGCCGTGAGTTTTGTTGCATTTAACTCCAATGGGGTTTTGCAAAATATTACGCATCCGTTGAATGATATTGACATCACCGTTAATAATACCGGTGTGTATGAAATTACGTATGAAGTAATGGCAATAAACACTAATGGTGGTATAGGCGTTAATAATAATGCTCAATTTTATTTAACTATTAATGGCGCAGATCAAAACGATACAAGAAATGGCGTTGGCTTTGGTGATGATCCAAATACACCACTTCAAATTCGTGGACAAGCAGTGTTTACCTTAAATGCGGGTGATATTGTTAATCTGGCTGCACGTACCGGTACCTTTAGTGTTGATCTTGTTCCACCACAGACAGGCTTTGCAGGATCGAGTATTAATGCCTCGATTTTAATTCGTCAAATCTCATAG

>Ppcl26

ATGGCAAGTTTAAACAAAGTAAGAGTTCAATTATTAGATACTATTACAGGAGCAGTACTACAAGAAGTTGATGTGCTAACAAGTGCGGATGCAGTTACATTCAGTGATGGACAAACATTTCAACAAAAACTAGACTCTGGATTATTAAAAGGAGCGACTGGTGCACAAGGAGTTCAAGGTATACAAGGTGAAGCGTTTTCTATATCTAAAGTATATAGTTCTGTTTCAGCTATGAACTCAGGATACGCTACTGATGGTGTTGCTCAAGGTAAATTCGTATTAATAGATACAGGAAATATAAATGATACAGATAATGCCAAATTATATGTTAAAGGAACTAGTTCATATACGTATTTAACAGACCTTAGTGGTGCTAATGGTATTCAAGGGCCTCAGGGTCCTCAAGGAATACAAGGAGTTCAAGGTATAAAGGGAGAAAATGGAGCAACTGGAAGCACTGGGGCAGTAGGTGCTACTGGAGCAAAGGGTGAAATTGGGACAAGAGGTTCAGTATGGTATAGTGGAACATTAATAACTGGAACAAGCACAACAGCTACAACATTTAGTGGTAGCGGCATAACTTCAGCCCTAGTCAATGACCAATATTTTAATACGAGTACAGCAAATGTATATGTATGTACAATTGATGGAAATGCAAATACAGCAAAGTGGGTATATTCAATTTGCTTAAAAGGAGCAACTGGCGCCACAGGTGCACAAGGAATACAAGGTGCAAAAGGAGATGTGGGCGCCACTGGTGCAACAGGAGAAACAGTAAGAGTTGGAACAACTTATTCTACAGCAACACAAGCAAATTTATTTTTTAAATTAGTATAG

>Ppcl28

GTGATTTTAAACTTATTTCCTCCTTGCGGTTTCCCCTCCGTATGTAATATCCCCATCATTACGGGACCCACGGGATTCACAGGACCCACGGGATTCACAGGACCTACAGGATCCACAGGACCTACGGGATTCACAGGATCCACAGGATCCACAGGACCTACGGGATTCACAGGATTCACAGGATTCACAGGACCCACAGGACCCACAGGACCCACGGGTGATCCAGGGGCTACAGGATCCACGGGTGGTACAGGGATTACAGGGATTACAGGGGCTACGGGGGCTACAGGCCCTGGTTTGAATACGGATGTGACTATAGTAGCGGGGGGAGGTGCCGACAACCAAATCATCACCCCTACCCCCAACCCAGGTCCAGGAGCTACCGGAACTGCTGTAATTCTACAAACCGGTAACGGCCAAATATACGGAAGCGGGGATATACTGCTCATTGGAACCACAGATATCCTACTACCGAGTACCGGAACCTATTTAATGTCCTTTCACATAGATGCTAACTACTCCACTACTACCGGTACTGCTCCTGCTGCCGGTGCGCAGGGCAGTTATGTAGCCTATTTCCGACAGTTCACAGCGGATTTTTTCTTCAATCAAATAGTTGCGTTCTGGGTTGGCCCCGCTCTATTCGGCGATGCATTTGATTCTTCTATAAGCAACACGGTTCTGGGTTGTGTTCGTGATATTCCACCACACGGACTCAACAACCACTTTCGTCTTGAGATACAATTTAGCAGCTTTAATCCAGCAGTACCGGTCAATCTTAATGTACAACTTCCATCCACCATGGTCACGATAACAAAATATAGCGATAGTATTTGCAGCTAA

>Ppcl29

ATGATTTTAAATTTATTTCCCCCTTGCGGTTTCCCCTCCATATGTAATATCAGCATCATTACAGGGCCCACAGGATTTACAGGACCTACGGGACCCACGGGATCCACAGGACCTACGGGACCCACGGGACCCACAGGACCCACGGGATTTACAGGATTCACAGGACCTACGGGACCCACGGGACCTACGGGTGATCCAGGGGCTACAGGATCCACGGGACCCACGGGGATTACAGGGATTACGGGGATTACAGGGATTACAGGGGTTACGGGGGTTACAGGGCCAACCGGTCCAGTCATAACAACAGACGTTACCGTAATTCCCTCCACTGCTGGAGACATCCAAACACTTGCTCCGAATACTACAACACCGATTACCCTAGTAACCCCTAGCGGGACAATATTTGGAAGTGGTGATGTCATTGTTACCACTACCGATATCATACTGCCTAGGACAGGTACCTATGTGGTGAGTTTTTCCCTGCAGGGTAGACTTGCCTCAAATGTTCCTGGCACCGCTGGAGATATTAGAGCTTTTATACTGCAATCCAACACAGGTGCATCCTTCACTCAGGCACTGGGGACCAATGGTGATTCATTGGGTGTTGCTACTGGTGCCGTCGTTCAACCCAATGTTCCTAGCTCTGGTATCAGTAGCACAGGCTTGGCTTGTGTGAGTGATACAGGTGGAGGAAATTTAAGCAATATTCTACAACTAATAGTAGCTTGGGCATCCGGCCCTCCGTTAGCGGGTATTCCTACTTCTCTGGATGTACTTCTTTTTGAAACTGTTGTTACCGTATTTCAATATTCCACTAATATTTGCATATCCCAGTAG

>Ppcl30

TTGCTCATTGGCGGTAATTTATTTGTAAACGGAACGATTATTCAACCGGCTCAGGCAAGCGTGAGTAGCGGTGGGGCAACGGGTCCTAATGGCGCAACGGGCCCTCAAGGTGCTACCGGACCCAATGGTCCTACAGGGCCAACAGGAGACACTGGGCAAACTGGATTTACCGGTTTTACCGGCAATCAAGGAGCAACAGGTGCAACGGGTAACACGGGCAATACCGGTCCGCAGGGCCAAACGGGCAATACAGGGTTTACGGGTAATGCCGGTGCACAAGGCGCAACGGGCAATACCGGTCCGGACACTTTTACTGGTTTGACTGGCGCGTCTGGTGTCTTGCTCGGAGCTGCCGCATCGTATGGTTATTTTTACACAACGGGCTTAGCAACGGGTGTATCAGGAACTATTTTAGCAGGAAGTCCGGCCACGTTTGCAGGCTCTTTGCCGGTAGTGACACCAGGACTTGTGCTTGGTGGTGGAGGTACTACCATTACTGTTGCAGCTGCCGGTACGTATAAACTAAGTTATATTGTGCAAGGGATCACGAGTAGCGTGTTTGGATTGCGCATAAACGGTGTTGACGATCCAACAACCGTTTTTGCCCAATCTTCGCTCAATGCACAAGATATAGGTGAGGTAATTATTACTCTTCCAGCAGGTGCAGTAGTTCAATTAATAAATCGTGATACTGCCAATTTGAGTGTTCTTAATTCTATGGGTGGCGATCAACAAGGAACTGCATTTTCACTGACGATAATTAGATTGAATTGA

>Ppcl33

ATGTCACGATCACAAAATAATATTATTAATTATGTCGATAATCGAATTAATAGATTATATGATAGAATAAACTCGGAAAGATTTTCCGCAATTATACCCGGACCAAGAGGTATAAAAGGTGAAAATGGTAAAATTGGATTAAAAGGTAATAATGGTGATAAAGGTAATAATGGAAACACAGGGCAAAAAGGTAATTTTGGTAATGATGGATTAAAAGGTGATAAAGGAGATTCTGGTAATGATGGATTAAAAGGTGATAAAGGAGATTTTGGAAATGATGGATTAAAAGGTAACAAAGGAGATCTTGGAAATGATGGATTAAAAGGTGATAAGGGAGAAGTTGGTAATGATGGATTAAAAGGTGATAAAGGAGATCTTGGAAATGATGGATTAAAAGGTGATATCGGATCAAAGGGTGATATCGGATCAAAGGGTGATACCGGATCAAAGGGCGAATCTGGAAGTACAGCAGAAAAAGGTGATAAAGGAAATGATGGTAATAAAGGAAATGATGGTAATAAGGGTGATAAAGGTATGCAAGGTAAAGGGTTTAAATATAGATGCAATTATGATCCATGTAAAATATATTATTATAACGATGTAGTAACTGTTAATACTTGTTGTTGCAATAGTATTTATGTATATACTTGTAAAACAAAAACAGGACCTTTATCAAGATGTACAAATATTTGTAATGTTCCTGGATGGAAATTAATGTTAAAAATATGCAATAAAAATCCATGTGATATAAATTCTGATTCATCAGATAGTGACTCATGTATTGATACATGTGTAAAACAATTAATGTGTGAAACAAATTATCCAAATATTAAAATAAATAATGATAACTATCGTAATATTTGTCTTCCAATATGCAACAATAATTATTTAGCAAAAAATAATTATCTAAAATATACTGGCATATGGCGCCGAAATTATTATTATGATATTGGAGATTTAACTATGTTAAATAATATTATATATATTGCTACAACTAATAATAATGATGAAATACCACATAATGGATCATTATATTGGGACGTATTTATTAATAATAATGTTATTTATCAAGGAATTTGGAAAATGCAAAAAACTTATTCAGTAAACAATATTGTTATACATAATGGATATACATATATATCTATTGATTATCCTCCGAAAGGAACCGATATTCAAAATCAAAATTATTGGGCCTTATTAAATTTTGATAATAATACAAATAATAATAATAATACAAATAAGGATAATAATATTATAGAAACATACAATGGAAGTGATATAATATCTTCTATTGATATAAATTCAAATGAATCATGTAGTATAATAAAATCACCATCAGATGCATTATATTACGCATATATTAACGAAGATGTATTAATACCTGCATTGAATTCACTTCATGTTTTTGATTTAAAATTTGAAAAAATTGAAAATAATATTAATATTATACCAGAAGAACAATATATAATATTTTTTAATCCAGGTACTTATAAAATAACTTTAAATATTAATTTTTCTGGAATTAATAATTTAACATCAAATGGTTATATATATGCCAAAAATAATTTGCAAGAAGTAAAAGAAATAATTCCAGCTAATAATGAAATTATTTTTAACAAAACATTAAATAATACTTTGCAACATATATTCCCCGTAACCATAACATATCCGAATTCTAAAATTTGGTTACGTATTAATTGGAGTAATCAAAGTGATATTATTACCATTAAATCTCAAAAAACCTGGATTTTAATAGAACAAATATATTAA
